# Benzimidazole, coumrindione and flavone derivatives as alternate UV laser desorption ionization (LDI) matrices for peptides analysis

**DOI:** 10.1186/1752-153X-7-77

**Published:** 2013-04-26

**Authors:** Syed Ghulam Musharraf, Aisha Bibi, Najia Shahid, Muhammad Najam-ul-Haq, Nida Ambreen, Momin Khan, Khalid Mohammed Khan, M Iqbal Choudhary, Atta ur Rahman

**Affiliations:** 1 Dr. Panjwani Center for Molecular Medicine and Drug Research, International Center for Chemical and Biological Sciences, University of Karachi, Karachi 75270, Pakistan; 2 H.E.J. Research Institute of Chemistry, International Center for Chemical and Biological Sciences, University of Karachi, Karachi 75270, Pakistan; 3 Department of Chemistry, Bahauddin Zakariya University (BZU), Multan 60800, Pakistan

**Keywords:** MALDI-MS, LDI matrix, Benzimidazole derivatives, Coumarin derivatives, Flavones, Peptides

## Abstract

**Background:**

Matrix-assisted laser desorption/ionization (MALDI) is a soft ionization mass spectrometric technique, allowing the analysis of bio-molecules and other macromolecules. The matrix molecules require certain characteristic features to serve in the laser desorption/ionization mechanism. Therefore, only a limited number of compounds have been identified as ultraviolet- laser desorption/ionization (UV-LDI) matrices. However, many of these routine matrices generate background signals that useful information is often lost in them. We have reported flavones, coumarindione and benzimidazole derivatives as alternate UV-LDI matrices.

**Results:**

Thirty one compounds have been successfully employed by us as matrices for the analysis of low molecular weight (LMW) peptides (up to 2000 Da). Two peptides, bradykinin and renin substrate tetra-decapeptide were analyzed by using the newly developed matrices. The MS measurements were made after mixing the matrix solution with analyte by using dried droplet sample preparation procedures. The synthesized matrix materials showed better S/N ratios and minimal background signals for low mass range. Furthermore, pico molar concentrations of [Glu^1^]-fibrinopeptide B human could be easily analyzed with these matrices. Finally, BSA-digest was analyzed and identified through database search against Swiss-Prot by using Mascot.

**Conclusions:**

These results validate the good performance of the synthesized UV-laser desorption/ionization (LDI) matrices for the analysis of low molecular weight peptides.

## Introduction

MALDI-MS was developed in the late 1980s and since then it has become a powerful tool for the analysis of large biomolecules, such as proteins, peptides, and nucleic acids, etc. [1]. Two important factors that involved in the ionization of analytes in the MALDI processing are laser and matrices. MALDI techniques commonly employ the use of UV lasers such as nitrogen lasers (337 nm) and frequency-tripled and quadrupled Nd: YAG lasers (355 nm and 266 nm, respectively) as a energy source. Moreover, radiation from IR laser such as Er: YAG lasers and CO_2_ are also employed [2]. Several modifications have been made on UV lasers to improve the performance. Recently, a modified laser named “smartbeam” has been introduced by the Bruker which is a combination of the speed of a solid-state-laser and the wide range of nitrogen lasers and provides substantial improvement in MALDI performance [3].

MALDI matrices are generally small organic compounds and act as energy mediators, effectively transferring the laser energy from a source to the surrounding sample molecules, resulting in minimum fragmentation. The method involves both laser ablation and ionization of the matrix/analyte mixture after electronic excitation of the matrix [4]. It is generally observed that effective LDI matrices have an aromatic conjugated system in their structures which enhance the energy absorbing capacity from UV laser. This phenomenon is still relatively unclear therefore certain molecule can act as matrices and others not [5]. Moreover, routinely used commercially available matrices such as HCCA (α-cyano-4-hydroxycinnamic acid), DHB (2,5-dihydroxybenzoic acid) and SA (sinapinic acid) also get ionized, producing large number of background signals in the low-mass region which restricted the use of MALDI for the determination of low molecular weight analyte. In the investigations of low molecular weight compounds, a number of new matrices and many modifications of the MALDI targets have been accomplished [6-25].

In an attempt to correlate the performance of a matrix in a MALDI experiment with its chemical structure, we studied thirty one synthetic alternate matrices for their desorption and ionization of intact small peptides. A large number of derivatives of flavones, coumarindione (coumarin derivative) and benzimidazol with necessary functionalities have been screened as potential matrices without any tedious chemical modification or applying expensive procedures.

## Experimental

### Chemicals and reagents

HPLC grade methanol, trifluoro acetic acid, acetone, and acetonitrile were purchased from Sigma-Aldrich (USA). All standards, ([Glu^1^]-fibrinopeptide B human (Glufib, ≥90%), bradykinin (≥97%) and renin substrate tetra-decapeptide (≥97%), were obtained from Sigma-Aldrich (USA). Cholic acid (≥97%) was obtained from Wako (Japan) while bovine serum albumin (BSA)-digest was purchased from Bruker Daltonics (Germany). Deionized water (Milli-Q) was used throughout the study (Millipore, USA). 2-Phenylenediamine, 4-hydroxy coumarin, ethyl benzoyl acetate, phloroglucinol and the other reagents and solvents used in the preparation of matrices were of synthetic grades, obtained from Sigma-Aldrich (USA).

### Preparation of matrix materials

Benzimidazole derivatives were synthesized by reacting together commercially available 2-phenylenediamine and different substituted aromatic aldehydes in *N*,*N*-dimethylformamide (DMF). The mixture was heated to reflux for 2 h; the progress of the reaction was monitored by TLC. After completion of the reaction, the mixture was allowed to cool to room temperature. Addition of water resulted in precipitation of a solid material, after filtration, the solid benzimidazole derivatives were obtained in high yields [26]. Coumarindione (coumarin derivative) were synthesized by taking a mixture of different substituted benzaldehyde and 4-hydroxy coumarin in water. Heating and stirring was continued for 1–2 h at 100°C. The reaction progress was monitored by TLC. On completion, the solid was filtered and washed subsequently with boiling water and hexane. After drying in vacuum, the coumarindione was obtained as a solid product. Flavones were synthesized by mixing different substituted aromatic β-ketoesters and substituted phenols, followed by irradiation with microwaves 300 W at 100°C for 3 min. The crude product was dissolved in 10% aq. NaOH and washed with diethyl ether. The product was precipitated by adding concentrated HCl, which was filtered, washed with water and vacuum-dried to obtain the desired flavones [27].

### Preparation of standard solutions

Peptide standards, bradykinin and renin substrate tetra-decapeptide, were dissolved in 0.1% TFA: ACN (1:1), while cholic acid was dissolved in methanol (1 mg/mL). The stock solution of peptide standards was prepared in a 1 mM concentration, and working standard solutions of 25 pM were prepared through the dilution of stock solution. BSA-digest was dissolved in 0.1% trifluoroacetic acid (TFA) in a concentration of 4 pM. [Glu^1^]-fibrinopeptide B human was used for sensitivity measurements in different concentrations of 1000, 100, 50, 25, 12.5 and 10 pM.

### UV–Vis absorption measurements

Synthesized materials of benzimidazole, flavones and coumarindione derivatives were dissolved in methanol having concentration of 1.0-0.5 mM. The absorption maxima was recorded by UV/Visible scanning in the region of 200–800 nm against the reagent blank on a UV/Visible double beam spectrophotometer (Thermo Scientific Evolution 300, UK), є and Log є were calculated at λ_max_ by Lambert-Beer law.

### Instrumentation

MALDI-MS measurements were carried out on Ultraflex III TOF/TOF (Bruker Daltonics, Bremen, Germany) mass spectrometer, equipped with a smartbeam laser (Nd: YAG, 355 nm, maximum pulse energy of 300 μJ, beam diameter 0.4 mm) and an electrostatic reflector. The focal length of lense is 50 mm. Mass spectra were recorded in reflector mode with the ion source 1 (ISI) set to 25.00 kV, source 2 (ISI) set to 21.50 kV, a lens voltage of 9.51 kV and performed with the laser energies of 55 ± 5%. The validation of data including the baseline subtraction was obtained through Flex analysis. External calibration was carried by using peptide calibration standards (Bruker Daltonics, Bremen,Germany). 0.1% TFA solution was prepared in deionized water and synthetic matrix materials were dissolved in different concentration (1–2 mM) in the solvent system of 0.1% TFA: ACN (1:1). The solution was sonicated for 5 minutes on a sonicator (Ultrasonic LC 38H), followed by centrifugation (Centrifuge 5804R Eppendorf). The supernatant was employed as a matrix. All measurements were made after mixing the matrix solution with analyte at a volume ratio of 1:2, spotted 1.5 μL droplet of mixture on the MALDI target plate (MTP 384, spot diameter 3.5 mm) and allowing the droplet to dry at ambient conditions. The dried droplet preparation method was used for the sample preparation. The dried co-crystallized sample was then analyzed by MALDI-MS. To improve the signal-to-noise ratio, spectral data from every 200 laser shots were summed. Then, the results from 3 different locations on a sample spot were summed. Hence, each point in such spectra corresponds to the summation of over 600 shots. The background spectra of matrices was recorded on off mode (provided in the Additional file 1: Figure S1) while with the analyte, the spectra were recorded on deflection mode in order to minimize all background signals generating from matrix.

Recorded BSA digest spectra with various matrices was submitted to the MASCOT search engine (Matrix Science, London, UK), using UniProt/Swiss-Prot (release July 2010, Homo sapiens, 18055 sequences) as the reference database. Mascot search parameters were as follows: enzyme specificity trypsin, fixed modifications cysteine carbamidomethylation, variable modification methionine oxidation. The maximum number of missed cleavages was set to 3 and mass tolerance at (0.1-0.9 Da). Stereomicroscopic image of matrices spots were recorded by using Nikon SMZ 800 (Japan) fitted with (DS-Vi1) camera.

## Results and discussion

Synthesized matrix materials of different classes of compounds, including benzimidazole derivatives (class I, fourteen compounds), coumarindiones (class II, twelve coumarin derivatives) and flavones (class III, six compounds) were evaluated as alternate matrices for the detection of low molecular weight peptides (Table 1). Wavelength maxima (λ_max_) of synthetic matrix materials were measured to verify their ability to absorb the laser energy at proper λ_max_ employed in ultraviolet-Matrix-assisted laser desorption/ionization mass spectrometry (UV-MALDI-MS). Spectrum quality generally increases with absorption [28], best mass spectrometric performances were observed at wavelengths near absorption maxima of matrices [4]. However, the desorption/ionization process is not solely dependent upon this relationship, as few good working routine matrices have λ_max_ quite far away from N_2_ laser, (377 nm), like nicotinic acid (260 nm), picolinic acid (266 nm), etc. [29]. Therefore, it is often believed that the matrices based desorption/ionization mechanism is still not completely understood. MALDI software generated signal intensities and S/N values (Table 2) also indicate this anomaly. For instance, compound 3 and 12 having the same λ_max_ i.e. 298 nm, the signal intensity of 12 is one of the highest in both cases of bradykinin and renin substrate tetra-decapeptide, while, intensity levels of compound 3 are among the lowest.

**Table 1:** **The λ**_**max **_**and molar absorptivities of different classes of compounds**

**S.N**	**Matrix classes**	**Structure**	**λ**_**max**_** (nm)**	**є**^**a**^	**Log є**
	**Class** (**I**) **Benzimidazole derivatives**				
**1.**	4-(1*H*-Benzimidazol-2-yl)phenol		299	19041	4.3
**2.**	5-(1*H*-Benzimidazol-2-yl)-2-ethoxyphenol hydrate		229	16437	4.2
**3.**	2-(3-Chlorophenyl)-1*H*-benzimidazole		298	10987	4.0
**4.**	2-(1-Naphthyl)-1*H*-benzimidazole		230	13705	4.1
**5.**	2-(3,4-Dimethoxyphenyl)-1-phenyl-1*H*- benzimidazole		223	24942	4.4
**6.**	2-(1*H*-Benzimidazol-2-yl)-5-hydroperoxyphenol		315	20238	4.3
**7.**	2-(4-Isopropylphenyl)-1*H*-benzimidazole		298	19812	4.3
**8.**	3-(1*H*-Benzimidazol-2-yl) phenol		213	7143	3.8
**9.**	2-(1*H*-Benzimidazol-2-yl)-4-chlorophenol		214	9313	4.0
**10.**	2-(4-Ethoxyphenyl)-1*H*-benzimidazole		298	14002	4.1
**11.**	*N*, *N*-Dimethyl-4-(1-phenyl-1*H*-benzimidazol-2-yl) aniline		325	28295	4.4
**12.**	2-(3,4-Dimethoxyphenyl)-1*H*-benzimidazole		298	15107	4.2
**13.**	2-(3,4-Dichlorophenyl)-1*H*-benzimidazole		309	17452	4.2
**14.**	2-(1*H*-Benzimidazol-2-yl)-1,4-benzenediol		213	13545	4.1
	**Class**-**11** (**Coumarin derivative**)				
**15.**	3-[(5-Methyl-2-furyl)methylidene]-2*H*-chromene-2,4-dione		213	16009	4.2
**16.**	3-[(2-Bromophenyl)methyl idene]-2*H*-chromene-2,4-dione		213	10209	4.0
**17.**	3-[(2-Nitrophenyl)methyl idene]-2*H*-chromene-2,4-dione		214	16985	4.2
**18.**	3-[(2,4-Dihydroxy phenyl)methylidene]-2*H*-chromene-2,4-dione		212	15120	4.2
**19.**	3-[2-Furylmethylidene]-2*H*-chromene-2,4-dione		214	16067	4.2
**20.**	3-[(2,3,4-Trihydroxyphenyl) methyl idene]-2*H*-chromene-2,4-dione		213	16720	4.2
**21.**	3-[(2-Ethoxyphenyl)methyl idene]-2*H*-chromene-2,4-dione		213	23618	4.4
**22.**	3-[(2-Hydroxy-3-methoxy phenyl)methylidene]-2*H*-chromene-2,4-dione		214	11682	4.1
**23.**	3-[(2-Fluorophenyl) methylidene]-2*H*-chromene-2,4-dione		213	13596	4.1
**24.**	3-[(3-Fluorophenyl) methylidene]-2*H*-chromene-2,4-dione		279	21981	4.3
**25.**	3-[(4-Hydroxyphenyl)methyl idene]-2*H*-chromene-2,4-dione		298	16720	4.2
**26.**	4-Methyl-2*H*-chromen-2-one		324	5779	3.8
	**Class**-**III** (**Flavones**)				
**27.**	7-Hydroxy-2-phenyl-4*H*-chromen-4-one		306	17642	4.2
**28.**	5-Methyl-2-phenyl-4*H*-chromen-4-one		261	15211	4.2
**29.**	5,7-Dihydroxy-2-phenyl-4*H*-chromen-4-one		268	22629	4.4
**30.**	2-(3,4-Dimethoxyphenyl)-4*H*-chromen-4-one		334	17175	4.2
**31.**	2-(3,4-Dimethoxyphenyl)-7-methyl-4*H*-chromen-4-one		334	14432	4.1

**Table 2:** **Signal intensity and S**/**N ratio of synthetic matrix material with low molecular weight analytes**

**Compounds**	**Bradykinin **^**a**^		**Renin substrate tetra-decapeptide **^**a**^	
	**S/N**	**Sig. Int.**	**S/N**	**Sig. Int.**
**1**	711	56530	551	47162
**2**	540	23276	423	23276
**3**	547	61687	228	23778
**4**	447	64184	205	50958
**5**	965	179163	579	39417
**6**	612	73058	450	40258
**7**	403	98986	586	132521
**8**	710	105850	689	68272
**9**	148	254785	435	32688
**10**	250	38696	411	59285
**11**	568	129858	361	80878
**12**	524	157277	360	144048
**13**	548	58187	620	105141
**14**	520	131703	668	127967
**15**	612	17858	402	55811
**16**	369	24724	463	65700
**17**	250	57522	432	81912
**18**	332	101983	299	61957
**19**	294	55402	200	13615
**20**	286	13022	514	14110
**21**	244	53297	446	36294
**22**	427	88683	642	101135
**23**	455	85541	650	53205
**24**	328	11451	519	81163
**25**	850	16992	574	85517
**26**	402	14593	436	79334
**27**	370	26798	444	66542
**28**	202	10398	221	13736
**29**	727	115238	309	124664
**30**	708	10452	125	16030
**31**	806	89386	66	18759
**HCCA**	832	86824	792	147702

^a^ 25pM.

### Method optimization

**Figure 1:**
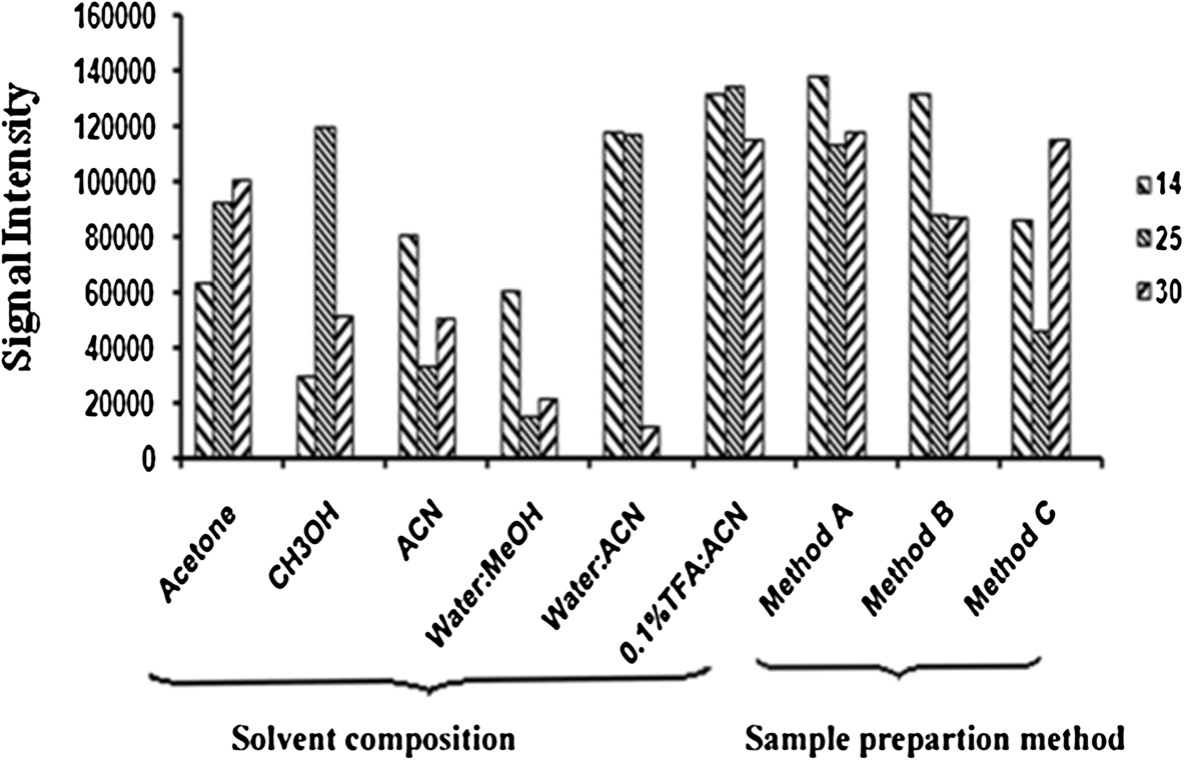
Solvent and method optimization of various matrices with bradykinin.

Two important parameters, including are sample preparation method and solvent selection, which played an important role in getting a better signal to noise ratio (S/N) [30,31]. One member of each class was used for optimization of the method before screening all the synthetic compounds. Sample preparation is one of the most important factors in MALDI-MS analysis. Three different commonly used sample preparation methods for laser desorption/ionization (LDI) were tried and compared by using different synthetic matrices, while 0.1% TFA: ACN was used as the suspending solvent. According to method A, the sample was mixed with synthetic matrix solution in a ratio of 1:2 and placed on a target plate. In method B, a thin layer of 1 μL of synthetic matrix material solution was made on a target plate and then 0.5 μL of analyte was added to it. Method C involves a similar strategy as that of method B, except that the synthetic matrix solution was placed on top of the sample matrix layer (sandwich method). Analysis of bradykinin as an analyte under three different conditions showed that method A was the best sample spotting method and it was therefore selected for the study (Figure 1). The preference of method A was based on the high signal intensity and better S/N ratio. Similarly, the solvent used for the solution/suspension of newly evolved matrix materials is another important factor in sample preparation. Different solvent systems, such as acetone, methanol, acetonitrile, (1:1) water/methanol, (1:1) water/acetonitrile, and 0.1% TFA: ACN were used. 0.1% TFA: ACN was selected due to the better results of the above-mentioned mass spectral parameters (Figure 1).

### Screening of compounds

All synthetic compounds were prepared for screening in 0.1% TFA: ACN with matrix to analyte volume ratio of 1:2 for the detection of low molecular weight peptides, bradykinin and renin substrate tetra-decapeptide (<2000 Da).

Fourteen compounds of class I (benzimidazole derivatives) possessing λ_max_ around 213–325 nm, were screened. All these new synthetic matrices of class I was comparable to existing MALDI matrix, HCCA in terms of intensity for the detection of peptides. However, the new matrices showed almost no background signals in the low mass range and hence were found to be suitable for the analysis of small peptides. Compounds 5, 8, 9, 11, 12 and 14 showed very high intensity (>1× 10^5^) for peptides (Table 2).

Twelve compounds of class II (coumarindione) were also screened as LDI matrix. All of them showed high intensity (>1× 10^4^) for low molecular weight peptides. Moreover, coumarin derivatives possess λ_max_ between 212–324 nm, most of the compounds showed best results. However, coumarin dyes [32] and 3-hydroxycoumarin [33] have been reported as a MALDI matrix for the analysis of proteins and oligodeoxynucleotides, respectively. Five compounds of class III (flavones), possessing λ_max_ between 261–334 nm, were also tested as LDI matrices. Both peptides showed good signal intensity (>1× 10^4^) as presented in Table 2.

**Figure 2:**
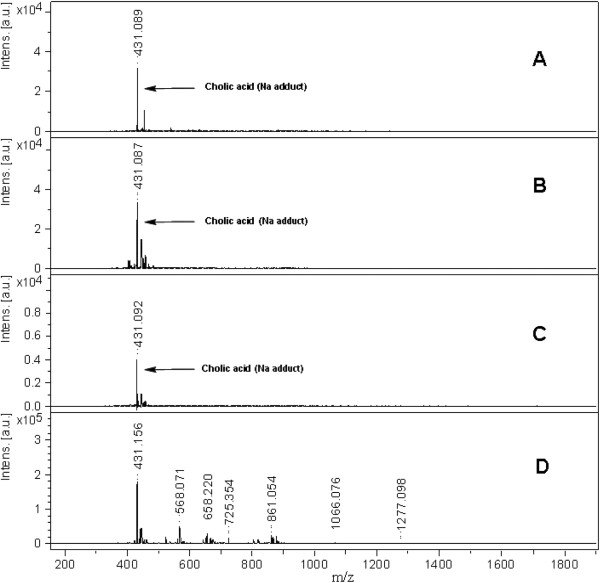
**MALDI mass spectra of cholic acid (M. wt = 408**.**58 Da, 0.1 μM), recorded in reflector mode by averaging 600 laser shots with smart beam (355 nm) on MALDI-TOF-MS by applying different compounds as a matrix (A) 6 (B) 25 (C) 29 (D) HCCA.**

**Figure 3:**
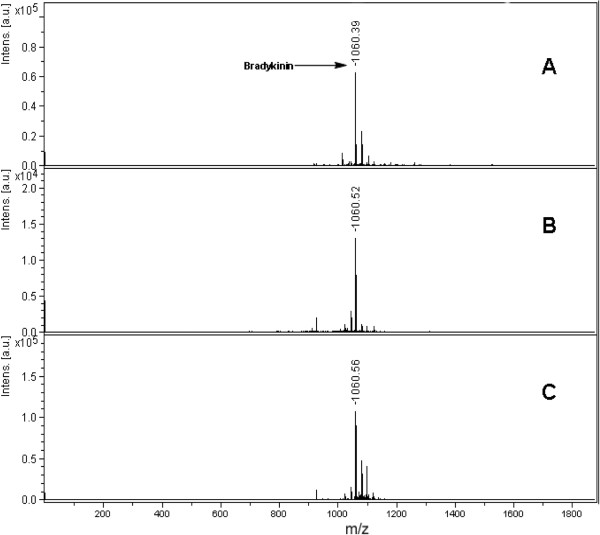
**MALDI mass spectra of a peptide bradykinin (25 pM), recorded in reflector mode by averaging 600 laser shots with smart beam (355 nm) on MALDI-TOF-MS by applying different compounds as a matrix (A) 6 (B) 25 (C) 29.**

Benzimidazole derivatives (1–14) posses different aromatic substitution at C-2. Aromatic ring substituted with electron withdrawing (halogen) and electron donating (−OH, -NR, -OR, R) groups at various positions. Compounds 6 and 14 which possess the same groups but at different orientations on phenyl ring i.e. *meta* and *para*, respectively, and it was found that *para* oriented OH groups exhibited better results. Moreover, in compound 12, when hydrogen at N-1 was substituted with phenyl ring as in compounds 5 then a decline in peptide signals were observed. In case of coumarins, compounds possessing furan ring (15 and 19) showed weak signals, whereas in dimethoxy substituted coumarin analogue (18) showed an enhanced signal in all coumarin derivatives. In flavone derivatives, dihydroxy containing flavone (29) showed high signal intensity as compare to compound 28 which lacks hydroxyl residues. Overall, signal intensities of analytes analyzed by newly developed matrices were promising. In all classes, different substituents were attached which to act as an auxochrome through electron lone pair on hetro atom and conjugated double bond system. Both n-π* and π-π* transitions enhanced the hyperchromic effect and lead to a bathochromic shift thus resulted an increase in wavelength. This means, less energy is required for the excitation. This factor is directly related to the amount of laser power required to get a good intensity signal from a sweet spot on MALDI target. There were a large number of interfering signals observed in lower mass range with HCCA whereas the spectra recorded with the new matrices were very clean with nominal interfering signals (Figure 2). 0.1 mM cholic acid solution was analyzed with three different matrices (compounds 6, 25 and 29) with a standard deviation of only ± 0.033, showing excellent reproducibility among all the matrices. The bradykinin peptide was analyzed with compounds 6, 25 and 29 with a standard deviation of ± 0.088 (Figure 3).

**Figure 4:**
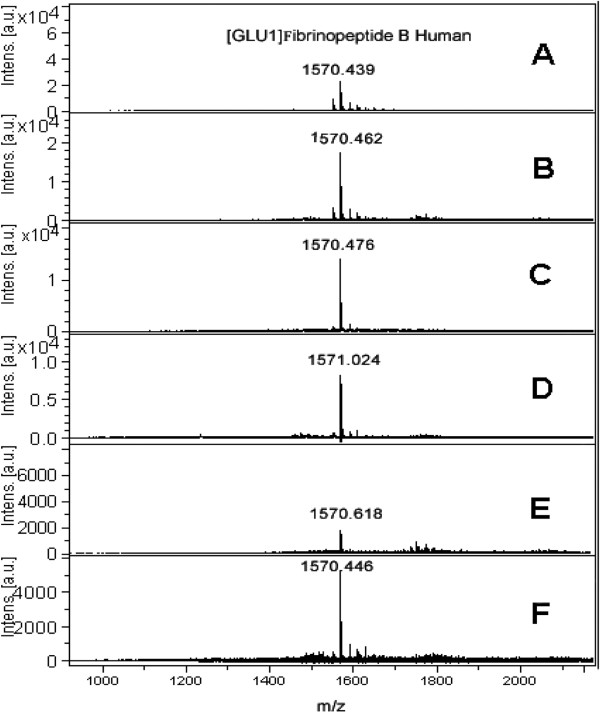
**Sensitivity measurement mass spectra of compound 29 with different concentration of [Glu1]-fibrinopeptide B Human recorded in reflector mode by averaging 600 laser shots with smart beam (355 nm) on MALDI-TOF-MS (A) 1000 (B) 100 (C) 50 (D) 25 (E) 12.5 (F) 10 pM.**

**Figure 5:**
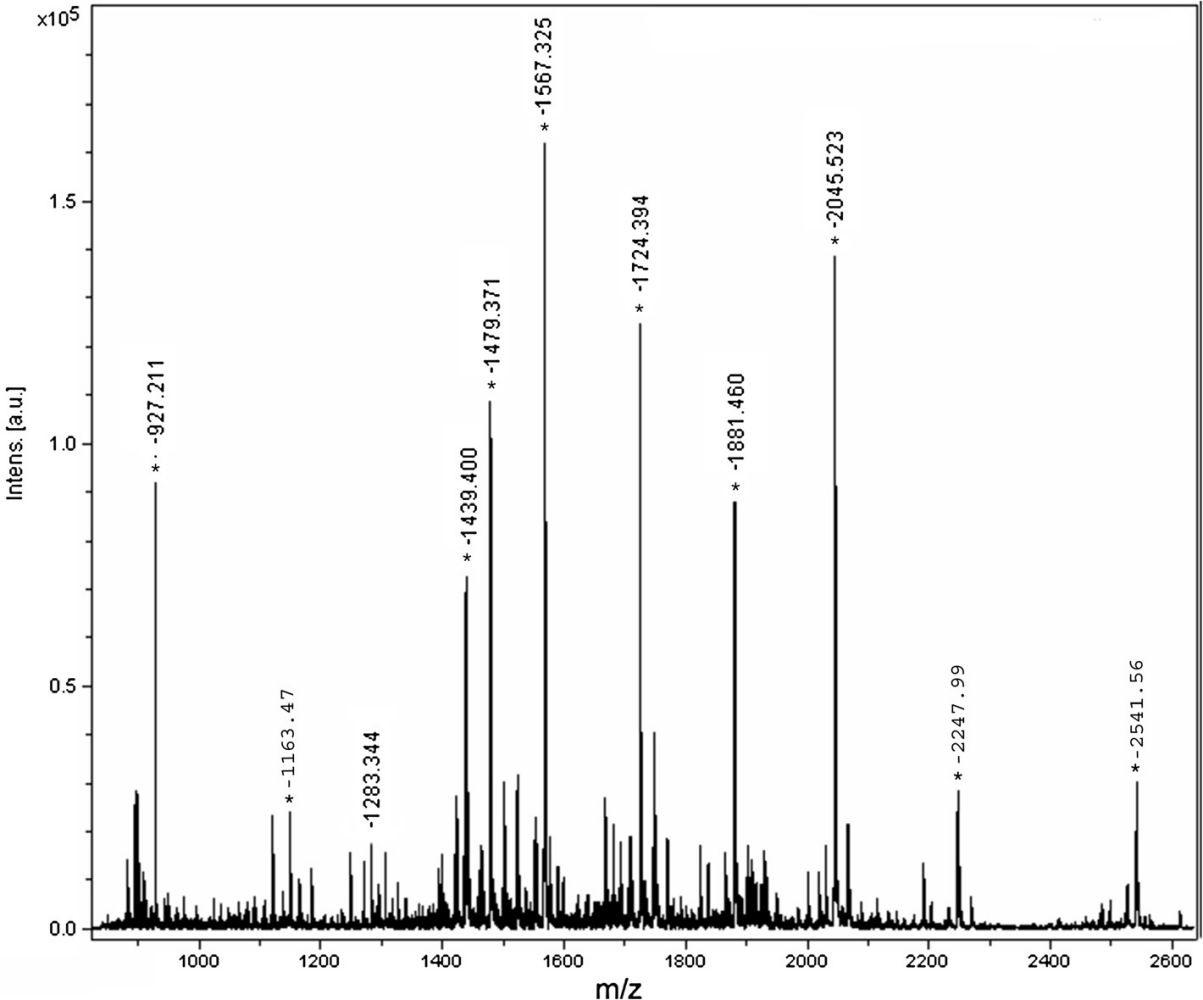
**MALDI mass spectra of BSA-digest with compound 6 recorded in reflector mode by averaging 600 laser shots with smart beam (355 nm) on MALDI-TOF-MS.**

Selected numbers of each class of compounds were subjected to various screening. Sensitivity measurement of [Glu^1^]-fibrinopeptide B was carried out by using compound 29 as an LDI matrix (Figure 4). Different concentrations of the analyte were screened including 1000, 100, 50, 25, 12.5 and 10 pM which showed intensity of 4 × 10^4^, 2 × 10^4^, 1.7 × 10^4^, 1.0 × 10^4^, 4.0 × 10^3^ and 3.0 × 10^3^, respectively. This unambiguously established the capability of new matrices to detect the less abundant analytes. Moreover, selected compounds from each class, including 6, 25 and 29, were also screened with BSA-digest sample which showed promising signals of peptides in the complex biological samples (Figure 5), assignment of significant peaks are mention in the Additional file 1: Table S1. The resulting mass spectrum was subjected to database search against Swiss-Prot, using MASCOT for the identification. The % sequence coverage of BSA using compound 6 was 53 and found to be close with HCCA 61 (Table 3), proving that the newly synthetic matrices posses ability to analyze peptides in complex biological samples.

**Table 3:** **Data**-**base search results of BSA**-**digest**, **analyzed with various matrices**

**Compounds**	**Average Mascot Score**	**Average number of Peptides**	**% Sequence coverage**
**6**	68	27	53
**25**	101	17	28
**29**	41	16	29
**HCCA**	81	35	61

**Figure 6:**
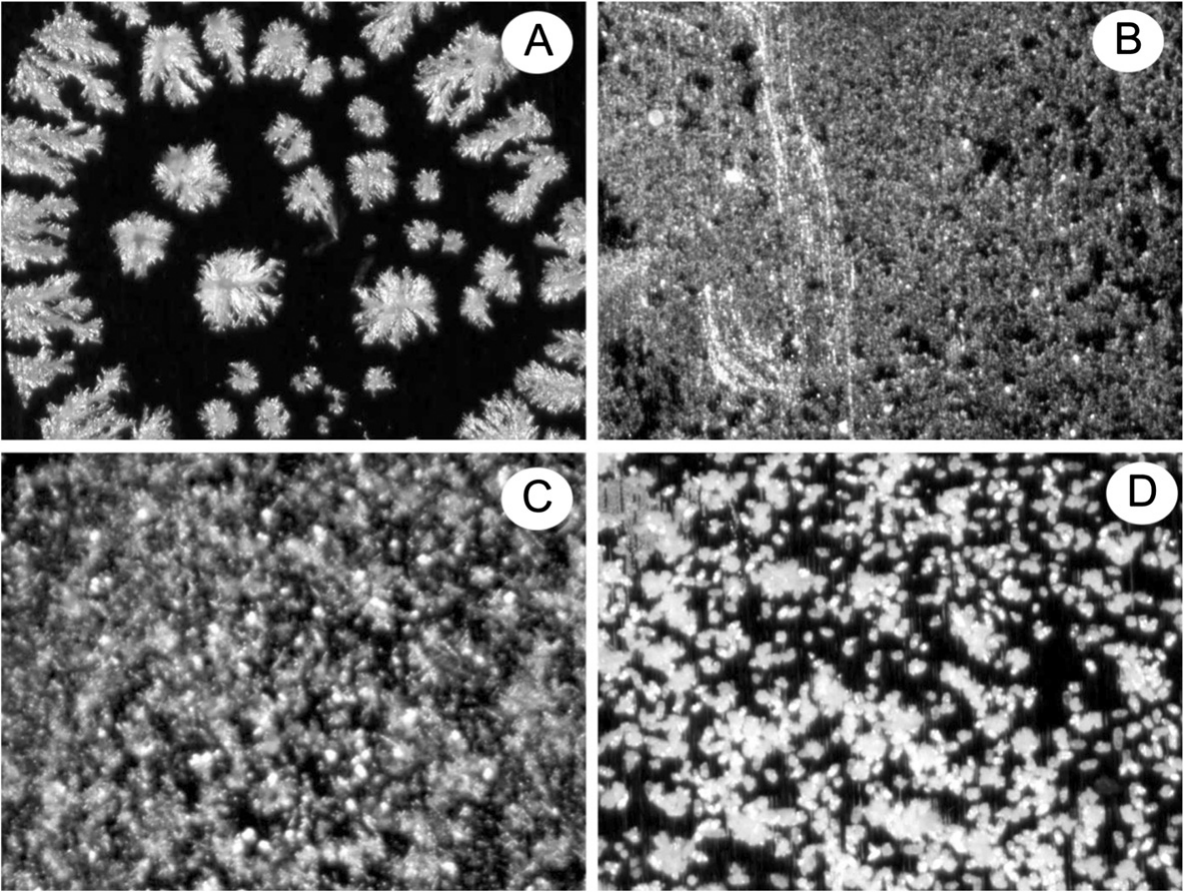
**Microscopic image of cocrystallized analyte of bradykinin with various matrices (A) 6 (B) 25 (C) 29 (D) HCCA.**

The homogeneity of newly synthetic matrices with the analyte solutions was also investigated. The achievement of the best spectral parameters (resolution, intensity and S/N ratio) is attributed to the homogeneous mixing/embedding of analyte molecules into the matrix molecules. This homogeneity makes the finding of sweet spots easier and is particularly useful near the detection limit ranges. Moreover, In MALDI-MS analyses, the matrix should co-crystallize homogeneously with the analyte, and this phenomenon is helpful when recording of MALDI-MS spectra is carried out in an automatic mode. Figure 6 shows the stereomicroscopic (SM) surface images obtained using different synthetic matrices materials that deposit on the sample target plate. The images showed homogeneous mixing and crystal formation of various matrices with the analyte samples and thus finding of the sweet spot is efficient and offers better reproducibility of results.

## Conclusion

Thirty one new matrices for laser desorption/ionization mass spectrometry (LDI-MS) have been studied and offered high performance and sensitivity which make them efficient platform for the analysis of small peptides. A number of conjugated aromatic systems in their structures ensure the laser energy transformations to the analytes. Furthermore, screening of a large number of organic compounds with various functionalities will be helpful for the development of structure–property relationship models, which in turn, could lead to a better understanding of the MALDI mechanism and discovery of better matrices in future.

## Competing interests

Authors declare that they have no competing interests.

## Authors’ contributions

SGM: Supervised the whole study and participated in method optimization. AB: Participated in performing experimental and manuscript preparation. NS: involved in performing experimental and manuscript preparation. MN: Involved in the useful discussion. NA: Synthesized class-1 compounds. MK: Synthesized class-II and class-III compounds. KMK: Supervised synthetic work. MIC: Involved in the discussion and manuscript checking. AR: Participated in manuscript checking. All authors read and approved the final manuscript.

## Supplementary Material

Additional file 1: Table S1 Peptide observed in tha MALDI TOF analysis of tryptic digestion of bovine serum albumin. **Figure S1.** The background spectra of matrices on off mode (**A**) compound 5 (**B**) compound 25 (**C**) compound 27. Click here for file
