# Supplementary material for: Benzimidazole, coumrindione and flavone derivatives as alternate UV laser desorption ionization (LDI) matrices for peptides analysis
Source: Chem Cent J. 2013 Apr 26;7:77. doi: 10.1186/1752-153X-7-77 (PMC3680071; doi:10.1186/1752-153X-7-77)
Supplement: Additional file 1: Table S1 — Peptide observed in tha MALDI TOF analysis of tryptic digestion of bovine serum albumin. Figure S1. The background spectra of matrices on off mode (A) compound 5 (B) compound 25 (C) compound 27. [file 1752-153X-7-77-S1.docx]

| **Measured molecular weight**  **(M+H)^+^ Da** | **Peptide sequence** | **Calculated molecular weight**  **(M+H)^+^ Da** |
| --- | --- | --- |
| 927.21* | YLYEIAR | 927.49 |
| 1139.20 |  |  |
| 1163.47* | LVNELTEFAK | 1163.63 |
|  | FKDLGEEHFK | 1249.62 |
| 1283.34* |  |  |
| 1305.50* | HLVDEPQNLIK | 1305.72 |
|  | TVMoENFVAFVDK | 1415.69 |
| 1439.40* | RHPEYAVVLLR | 1439.81 |
| 1479.37* | LGEYGFQNLIVR | 1479.80 |
| 1567.32* | DAFLGSFLYEYSR | 1567.74 |
|  | KVPQVTPTLVEVR | 1639.94 |
| 1724.39 |  |  |
| 1881.00* | RPC*FSALTPDETYVPK | 1880.82 |
|  | LFTFHADIC*TLPDTEK | 1907.92 |
| 2045.52* | RHPYFYAPELLYYANK | 2045.03 |
| 2247.99* | EC*C*HGDLLEC*ADDRDLAK | 2247.94 |
| 2541.56* | QEPERNEC*FLSHKDDSPDLPK | 2541.17 |
|  |  |  |

**Additional data files**

**Table S1** peptide observed in tha MALDI TOF analysis of tryptic digestion of bovine serum albumin

The masses denoted with asterisk (*) are the peptide masses matched by the search algorithm to the data base sequence. The other masses for which a peptide sequence is given are additional matches recognized by manual inspection of the database sequence and the mass spectral data**.**

**
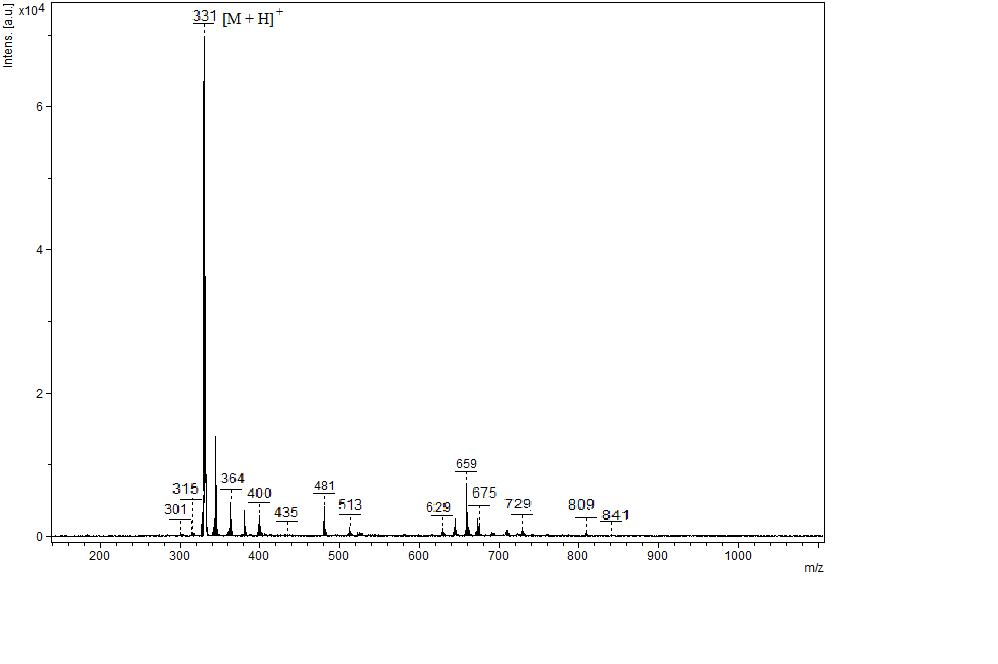
**

**A**

**
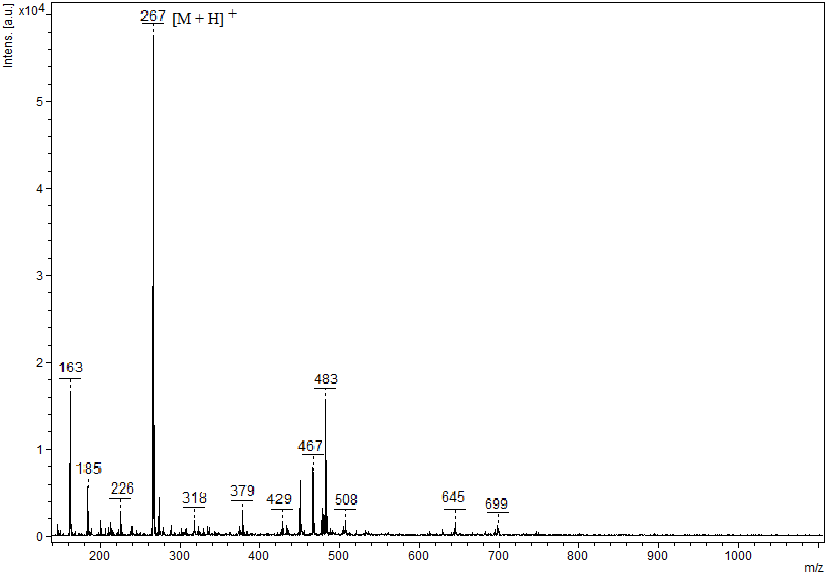
**

**B**

**
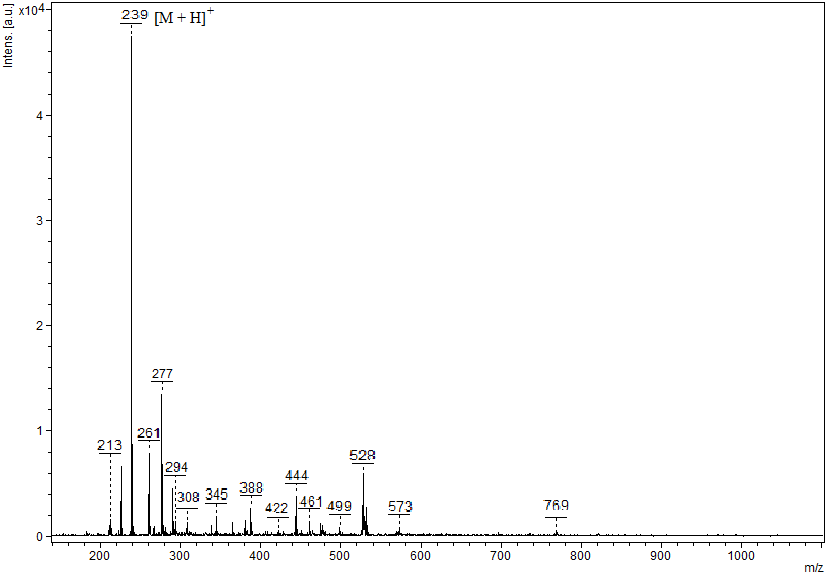
**

**C**

**Figure S1:** The background spectra of matrices on off mode (A) compound **5** (B) compound **25** (C) compound **27**
